# Supplementary material for: The regulation of hsacirc_004413 promotes proliferation and drug resistance of gastric cancer cells by acting as a competing endogenous RNA for miR-145-5p
Source: PeerJ. 2022 Apr 7;10:e12629. doi: 10.7717/peerj.12629 (PMC8995023; doi:10.7717/peerj.12629)
Supplement: Supplemental Information 2 [file peerj-10-12629-s002.docx]

The regulation of hsacirc_004413 promotes proliferation and drug resistance of gastric cancer cells by acting as a competing endogenous RNA for miR-145-5p

**The regulation of hsacirc_004413 promotes proliferation and drug** **resistance of gastric cancer cells by acting as a competing endogenous RNA for miR-145-5p**

Fusheng Zhoua ^1^，Weiqun Dingb^2^，Qiqi Maoa^1^, Xiaoyun Jianga^1^, Jiajie Chena^1^, Xianguang Zhao^1^, Weijia Xua^1^, Jiaxin Huanga^1^ ,Liang Zhonga^1^, Xu Sun ^1^

^1^Department of Gastroenterology, Huashan Hospital North, Fudan University, Shanghai, China

^2^ Department of Gastroenterology, Huashan Hospital, Fudan University, Shanghai , China

Corresponding Author:

Xu Sun ^1^

No.108, LuXiang Road,Shanghai , 201907, China

Email address: sunxu6060@163.com

Table 1. The sequence of siRNA.

| si-Circ hsacirc_004413 | sense | antisense |
| --- | --- | --- |
| si-Circ-1: | 5’-CCUGGGAAAGGCUUAUAACTT-3’ | 5’- GUUAUAAGCCUUUCCCAGGTT-3’ |
| si-circ-2 | 5’-GGGAAAGGCUUAUAACCCATT -3’ | 5’-UGGGUUAUAAGCCUUUCCCTT-3’ |
| Negative control | 5’-UUCUUCGAACGUGUCACGUTT-3′ | 5’- ACGUGACACGUUCGGAGAATT-3’ |
